# Supplementary material for: HIVprotI: an integrated web based platform for prediction and design of HIV proteins inhibitors
Source: J Cheminform. 2018 Mar 9;10:12. doi: 10.1186/s13321-018-0266-y (PMC5845081; doi:10.1186/s13321-018-0266-y)
Supplement: Supplementary file 2 — Additional file 2. Source code of HIVProtI web server. [file 13321_2018_266_MOESM2_ESM.zip › HIVprotI_Source-code/hivprotI/weka-3-6-6/doc/weka/core/stemmers/Stemming.html]

Stemming


---


|  |  |  |
| --- | --- | --- |
| **Overview** \| **Package** \| **Class** \| **Tree** \| **Deprecated** \| **Index** \| **Help** \| **Weka's home** | |  |
| **PREV CLASS**   NEXT CLASS | **FRAMES**    **NO FRAMES**     **All Classes** |  |
| SUMMARY: NESTED \| FIELD \| CONSTR \| METHOD | DETAIL: FIELD \| CONSTR \| METHOD |  |


---


## weka.core.stemmers Class Stemming

```
java.lang.Object
  weka.core.stemmers.Stemming
```

**All Implemented Interfaces:**: RevisionHandler

---

``` public class Stemming extends java.lang.Object implements RevisionHandler ```

A helper class for using the stemmers. Run with option '-h' to list
all the available options.

**Version:**
:   $Revision: 1.3 $

**Author:**
:   FracPete (fracpete at waikato dot ac dot nz)

---

**Constructor Summary**

`Stemming()`


| **Method Summary** | |
| --- | --- |
| `java.lang.String` | `getRevision()`             Returns the revision string. |
| `static void` | `useStemmer(Stemmer stemmer, java.lang.String[] options)`             Applies the given stemmer according to the given options. |

**Methods inherited from class java.lang.Object**

`equals, getClass, hashCode, notify, notifyAll, toString, wait, wait, wait`

**Constructor Detail**

### Stemming

```
public Stemming()
```


**Method Detail**

### useStemmer

```
public static void useStemmer(Stemmer stemmer,
                              java.lang.String[] options)
                       throws java.lang.Exception
```

:   Applies the given stemmer according to the given options. '-h' lists
    all the available options for the given stemmer.

    :   **Parameters:**: `stemmer` - the stemmer to use: `options` - the options for the stemmer **Throws:**: `java.lang.Exception` - if something goes wrong

---


### getRevision

```
public java.lang.String getRevision()
```

:   Returns the revision string.

    :   **Specified by:**: `getRevision` in interface `RevisionHandler`
    :   **Returns:**: the revision


---


|  |  |  |
| --- | --- | --- |
| **Overview** \| **Package** \| **Class** \| **Tree** \| **Deprecated** \| **Index** \| **Help** \| **Weka's home** | |  |
| **PREV CLASS**   NEXT CLASS | **FRAMES**    **NO FRAMES**     **All Classes** |  |
| SUMMARY: NESTED \| FIELD \| CONSTR \| METHOD | DETAIL: FIELD \| CONSTR \| METHOD |  |


---
